# Feasibility in a homeopathy for seasonal allergic rhinitis RCT: importance of therapeutic relationship and organizational capacity

**DOI:** 10.3389/falgy.2025.1694531

**Published:** 2026-01-15

**Authors:** J. Siewert, L. Joschko, R. Schleicher, B. Stöckigt, M. Teut, S. N. Willich, B. Brinkhaus, E. Jansen

**Affiliations:** 1 Institute of Social Medicine, Epidemiology and Health Economics, Charité – Universitätsmedizin Berlin, Corporate Member of Freie Universität Berlin and Humboldt-Universität zu Berlin, Berlin, Germany; 2 Institute of Social Medicine and Epidemiology, Medizinische Hochschule Brandenburg Theodor Fontane, Brandenburg an der Havel und Neuruppin, Brandenburg an der Havel, Germany

**Keywords:** complementary and alternative medicine, homeopathy, allergy, feasibilities studies, therapeutic relationschip

## Abstract

**Introduction:**

Seasonal allergic rhinitis (SAR) patients often use homeopathy for symptom relief. The HOMEOSAR trial, a randomized, placebo-controlled, triple-blind multicenter study, included a qualitative sub-study to assess feasibility. This sub-study specifically examined the feasibility of the study, focusing in particular on two mechanism-rich domains—the therapeutic relationship and study organization—with special attention to study procedures, physician–patient relationships, organizational aspects, and interactions with the study team.

**Methods:**

Semi-structured interviews with trial participants were conducted 6–8 weeks after baseline, corresponding to 2–4 weeks after study intervention ended. Data were coded, categorized, and analyzed using qualitative content analysis, both inductively from the material and deductively based on study aims. MAXQDA® software was used to support analysis.

**Results:**

Fifteen patients (mean age 43 years; range 20–67; 11 male) participated in the qualitative study (*n* = 8 standardized homeopathy, *n* = 2 individualized homeopathy, *n* = 5 placebo). They were recruited from nine study centers in Berlin. Results of the qualitative sub-study indicate the overall feasibility of the HOMEOSAR trial. Patients highlighted the empathetic and professional relationships with physicians and the supportive contact with the study team. While the documentation was seen as well-structured and clear, it was also described as time-consuming.

**Conclusion:**

Findings of this qualitative study provide design-level recommendations (e.g., upfront communication of consultation length, digital diaries with free-text, tracked medication shipping, single contact point) for RCT studies. Beyond the specific homeopathy study context, these findings offer important methodological insights for designing patient-centered RCTs in complex intervention settings not only in the field of Complementary and Integrative Medicine.

## 1 Introduction

Allergic rhinitis (AR) is a prevalent global health issue, affecting between 10% and 40% of the population worldwide (Brożek et al.). In the United States, the “Burden of Allergic Rhinitis in America” survey reported that physician-diagnosed AR had a prevalence of 22.0% (1). In Germany, the Versorgungsatlas reported that in 2019**,** 7.1% of individuals with statutory health insurance had a diagnosis of hay fever (2) while the German Health Interview and Examination Survey for Adults (DEGS1) documented a lifetime prevalence of 14.8% for hay fever in adults in 2013 (3). In addition, the International Study of the Allergic Rhinitis Survey reported AR prevalence rates of 15% to 25% across four world regions, with symptoms most pronounced in spring (51.9%) and autumn (28.9%) (4).

Conventional treatments for SAR include antihistamines, corticosteroids, decongestants, and leukotriene antagonists, which relieve symptoms but may cause side effects such as drowsiness, dry mouth, and headaches (5). This may impair quality of life and prompt patients to seek complementary medicine treatments (6, 7, 16). Specific immunotherapy (SIT), or hyposensitization, can desensitize the immune system but is time-consuming, costly, and not effective for all patients (8, 9). In Germany, a population-based case-control study found that 26.5% of participants with allergic diseases used complementary and alternative medicine, with 35.3% of them choosing homeopathy (10–12).

Several studies have explored the use of homeopathy for allergic rhinitis (AR), however the evidence for its efficacy is still limited (13–21), leading to an ongoing debate about its efficacy in treating the condition [see, for example (22–24, 42)].

Designing rigorous randomized, placebo-controlled trials in homeopathy is challenging, as recruitment difficulties and individualized treatment approaches often result in small sample sizes and complicate standardization and blinding procedures (23). As homeopathic treatment is individualized, standardized protocols are difficult to establish, and studies suggest that the time required for case-taking and the holistic consultation process may affect outcomes (25–27).

Recent qualitative research has shown that homeopathic consultations are perceived by practitioners as a complex, interconnected process in which remedy selection is embedded within broader therapeutic elements such as establishing connection, understanding the patient's journey, and fostering self-reflection (26). Another qualitative study described the decision-making process in homeopathy through a model that combines pattern recognition, hypothetico-deductive reasoning, and intuition to arrive at a remedy match, while also emphasizing the role of the patient–practitioner relationship and the need to minimize bias (25).

The quality of the relationship between homeopathic practitioners and patients plays a crucial role in the success of treatment, which presents challenges in upholding the strict standards required for clinical studies. A 2011 study on rheumatoid arthritis found that positive clinical effects were mainly attributable to the homeopathic consultation rather than the remedy itself (25). There is a debate as to whether treatment expectations can be influenced by various factors (including communication, setting, patient-centered communication style, rituals, empathy) and whether these factors are more prevalent in homeopathic treatment (28). In addition, there is a need for more high-quality, randomized controlled trials to better understand the potential benefits and limitations of homeopathy, also for the treatment of SAR. Feasibility of homeopathic treatment for SAR is increasingly discussed as a complementary approach to conventional care. To evaluate the process of the quantitative trial and integrate contextual factors, a qualitative interview-based component was incorporated into the study design (29). The quantitative HOMEOSAR trial, conducted as a triple-blind, placebo-controlled study, contributes to strengthening the evidence base in homeopathy, where such high-quality research is still limited. In line with guidance on feasibility research, this qualitative component complements the semi-explorative RCT design (30).

This study adds novelty by focusing on patient experiences, adherence, and consultation processes as contextual factors in homeopathy for SAR. The qualitative sub-study aimed to explore feasibility beyond quantitative outcomes. Feasibility in this context refers to the practical implementation of the intervention, the acceptability of procedures from a patient perspective, and the organizational capacity to conduct the trial as designed. The focus was on implementation and context-related factors such as physician-patient relationship, study organization, and team interactions.

## 2 Methods

### 2.1 Study background and participant recruitment

The qualitative analysis is based on a sub-study of the HOMEOSAR trial, a randomized, placebo-controlled, triple-blind, three-armed phase IV clinical trial investigating the efficacy of homeopathic drug treatment in patients with seasonal allergic rhinitis (SAR). The trial compared individualized homeopathic treatment (potency D6) and standardized homeopathic treatment with *Galphimia glauca* (potency D6) to placebo, all in addition to on-demand medication. The full study design and interventions are described elsewhere (29).

The aim of the qualitative component was to explore the feasibility of the HOMEOSAR trial from the patients’ perspective, particularly with regard to the practical implementation of the interventions, acceptability of study procedures, and contextual factors such as physician–patient interaction, structural conditions, and expectations.

Participants for the interview study were recruited from nine Berlin study sites. The recruitment was conducted via telephone by the study secretariat using a database-generated list of individuals who had completed the 8-week study protocol and returned at least four questionnaires. A total of 15 participants were included. Because the qualitative team was blinded to allocation, sampling did not use treatment-arm information; after all interviews were completed and unblinding for reporting, the allocation among interviewees was: standardized *Galphimia glauca* D6 (*n* = 8), individualized homeopathy (*n* = 2), and placebo (*n* = 5). The sample was skewed toward male participants (73%) and contained relatively few individualized homeopathy cases (*n* = 2).

### 2.2 Sampling strategy and recruitment process

We applied purposive sampling to maximize heterogeneity independent of treatment allocation (which was unknown to the qualitative team under blinding). We did not sample by arm. The study secretariat contacted eligible participants by telephone and asked for their willingness to participate in a semi-structured interview. Interview appointments were coordinated by the qualitative research team to maintain blinding between the qualitative team and allocation information. The recruitment process continued until a total of 15 participants had completed interviews, with variation sought in site, gender, age, baseline burden, and prior treatment experience. This number of interviews was considered sufficient to achieve adequate coverage of core themes (information power) across the range of participant perspectives.

### 2.3 Blinding

The parent RCT used concealed allocation and identical study preparations. Participants, treating physicians, site staff and statisticians were blinded to treatment assignment throughout the trial. For the qualitative sub-study, the interviewers and analysts remained blinded during recruitment, interviewing, and initial coding; they had no access to allocation information. Unblinding occurred only after all interviews had been completed and the initial codebook established, for the sole purpose of transparent descriptive reporting of the qualitative sample's distribution across trial arms.

### 2.4 Timing and conduct of interviews

Interviews were conducted between June 2022 and October 2023 by telephone. The interview duration ranged from 11 to 38 min, with an average of approximately 23 min. Interviews were carried out by trained researchers without any prior relationship to the participants. Participants were informed about the interviewer's role and the purpose of the qualitative sub-study before the interview. The interviews were conducted using a semi-structured approach and were guided by a thematic interview guide (see Supplementary Appendix 1). The guide addressed participants' experiences with the intervention, perceived effects, the physician-patient relationship, and organizational factors related to the study. As interviews were conducted 2–4 weeks after the end of treatment, there is a potential for recall bias regarding immediate treatment experiences.

### 2.5 Data collection procedure

The interviews were conducted by telephone, audio-recorded, and transcribed verbatim. Personal identifying data were pseudonymized. Sociodemographic descriptors for interviewees (e.g., age, sex, employment/occupation) were retrieved from the parent RCT's baseline case report forms. To protect confidentiality in this small qualitative subsample, we did not report specific occupations; where used, variables were collapsed to high-level categories (e.g., employed vs. not employed) and were used for descriptive purposes only. Linkage between the qualitative ID list and the RCT database was performed by the trial secretariat; the qualitative team received de-identified extracts only. Transcripts were not returned to participants for comment or correction; thus, no formal member checking was conducted. To counterbalance this, reflexive team discussions were used to enhance interpretive consistency. All interviews were conducted in accordance with ethical and data protection standards.

### 2.6 Interview content and guide development

The semi-structured interview guide (see Supplementary Appendix 1) was developed based on existing literature, the study protocol, and expert consultation. Key topics included perceived medical effects of the intervention, personal experience with the treatment, quality of life, and stress management. As part of the process evaluation, the interviews also addressed the study structure, contact with the study staff, and perceptions of study bureaucracy and documentation.

### 2.7 Data protection and transcription

All audio recordings were securely stored in encrypted format and deleted from the recording device after transfer. Transcripts were anonymized and retained in accordance with institutional data protection policies and applicable legal standards.

### 2.8 Data analysis

Data were analyzed using qualitative content analysis following Hsieh and Shannon (31). We employed a hybrid inductive–deductive qualitative content analysis: feasibility domains (e.g., acceptability, implementation, organizational capacity) acted as sensitizing concepts only; codes and sub-themes were developed inductively from the data using constant comparison. We explicitly avoided one-to-one mapping from interview questions to themes and checked emerging interpretations against deviant cases. The coding process combined inductive and deductive approaches based on the interview material and study aims. Coding and analysis were conducted using MAXQDA®; the software was used to support analysis (coding, data management, retrieval), with analytic decisions made by the research team. In a first step, meaningful units were identified and coded. These codes were grouped into descriptive categories and refined through iterative comparison to develop overarching themes that reflect participants’ experiences and perceptions. The analytic process was discussed regularly within the qualitative research team to ensure reflexivity and interpretive consistency throughout. The analysis team consisted of researchers with backgrounds in medicine, health science and sociology, none of whom had been involved in patient care during the RCT, which helped minimize role-related bias. Reflexivity was further addressed by documenting analytic decisions and critically reflecting on the influence of researchers' professional backgrounds during team discussions.

### 2.9 Ethical considerations

The study was approved by the Ethics Committee of the Berlin State Office for Health and Social Affairs (Landesamt für Gesundheit und Soziales Berlin, EudraCT-No.: 2019-003255-10) in March 2020. All participants provided written informed consent prior to the interview. The study was conducted in accordance with the Declaration of Helsinki and applicable data protection legislation. The design, conduct, and reporting of the qualitative sub-study followed the Consolidated Criteria for Reporting Qualitative Research (COREQ) checklist to ensure methodological rigor and transparency (32).

## 3 Results

### 3.1 Participants

A total of 15 participants took part in the qualitative interviews (see Table 1). The average age was 43 years (range: 20–67), with 11 male and 4 female participants. After unblinding, it was revealed that eight participants had received standardized homeopathy, two individualized homeopathy, and five placebo treatment. Most participants were employed (12/15; 80%).

**Table 1:** Sample characteristics (*N* = 15).

Variable	Value
Age in years, mean (range)	43 (20–67)
Sex (male/female), *n* (%)	11 (73%)/4 (27%)
Groups (standardized, individualized, placebo)	8, 2, 5
Employed (yes, no)	12, 3

However, the qualitative analysis revealed no relevant group differences in the subjective experiences or perceived effects of the interventions. Therefore, the following results are presented collectively, with a focus on participants' shared perspectives and cross-cutting themes.

### 3.2 Qualitative analysis results

The interviews were categorized into five main themes based on the qualitative analysis: (1) motivation to participate, (2) expectations of the medication, (3) therapeutic relationship, (4) organization of the study and contact with the study team, and (5) effect of the intervention (see Table 2). This paper focuses on two of the main categories and their sub-categories.

**Table 2:** Main and Sub-categories of the qualitative analysis.

Main category	Subcategory
1) The therapeutic relationship	Professionalism of the physician Inclusion of the individual biography Time intensity Empathy of the physician
2) Organization of the study and personal contact with the study team	Confusion in the organization Supportive study team Extensive paperwork Well-structured questionnaires Unclear expectation of diaries

For the purpose of this paper, we focus on two main categories: (1) the therapeutic relationship and (2) the organization of the study, including personal contact with the study team. These categories were selected because they emphasize interpersonal and procedural factors that are central to feasibility and have received less attention in previous homeopathy trials. The remaining categories—motivation to participate, expectations toward the medication, and perceived effects—are not analyzed in depth here, as they relate more closely to treatment effectiveness rather than to feasibility. Synthesized summaries of these additional themes are provided in Supplementary S1 to complement the main feasibility domains presented in this paper.

### 3.3 The therapeutic relationship

In the case of this study, physician contact occurred during the medical history assessment and at one follow-up visit. Most patients described their personal interactions with the study physician as professional, well-structured, and pleasant. Some evaluated it as “good”, and that there was “nothing to complain about”. One patient shared what they appreciate about the case-taking with the physician as follows:

“Definitely the pleasant atmosphere with the physician, she was quite calm and .. I think she was definitely very professional.’ (participant 1, standardized group, line 102)

Patients noted that the physician showed genuine interest in their personal histories, enabling a comprehensive exploration of their health concerns through in-depth questions, even about the onset of the SAR. Some patients described the case-taking as “holistic” because the physician inquired about their life circumstances and career history.

“At first I was a bit surprised because I didn't realize that it went so (deep), she (the physician) actually asked questions about my childhood and when it happened and what it was like. And I didn't expect that at all, so I was surprised.” (participant 2, placebo group, lines 46–49)

Most patients mentioned that the interview was time-intensive, which impacted their relationship with the physician and their ability to engage and build trust. Some expressed mild confusion, as they were accustomed to shorter interviews with other physicians, where they felt more like they were being treated as part of a “routine process” or “on an assembly line.” Others noted that this distinction exemplifies the difference between conventional medicine and homeopathy, as homeopathic physicians dedicated more time to taking a thorough case-taking.

'She took a lot of time when you needed it. And that's why I got on very well with her and also felt very well looked after' (participant 3, placebo group, lines 36–37)

Patients consistently highlighted the physicians' empathy and respectful demeanor, emphasizing their ability to create a calm and trusting atmosphere during the case-taking phase. Most patients mentioned that this fosters a foundation of trust and encourages them to share details of their medical history without feeling rushed, all within a calm and unhurried environment.

“It (the anamnesis interview) was very focused, it was very calm, in the sense of unexcited, which I found very good. There was also enough time to clarify everything, there was always the opportunity to ask questions or clarify anything that was unclear, it was also well introduced, it was (..) well structured.” (participant 4, placebo group, lines 112–117)

This sense of attentive listening and structured dialog promoted confidence in the treatment process among patients. However, it is also noted that the bureaucratic requirements of the study sometimes required self-organizing skills but did not negatively impact personal contact.

One patient described it as: “I thought to myself, what's it going to be now? Because I thought it would be a first interview, well, uh, all the bureaucracy, but I thought I was in good hands.” (participant 5, individualized group, line 107).

### 3.4 Organization of the study and personal contact with the study team

Seven patients expressed confusion about the study's organization. Two patients mentioned experiencing organizational difficulties related to structural ambiguities in appointment management. They reported that it was not clearly communicated how long the initial homeopathic consultation would take, and they found it unexpectedly lengthy compared to their prior experiences with other physicians. Additionally, two patients encountered confusion regarding logistical details, such as the time and location of the anamnesis interview—they initially received the wrong address but eventually managed to find the correct one. Furthermore, two patients reported issues with receiving their medication via post, including delays in delivery.

“I think it worked as far as possible, as I said, only the study drug, nobody knew where it ended up” (participant 6, standardized group, line 121)

Nevertheless, the study team supported patients in overcoming these organizational challenges and is described as accessible and helpful. Patients felt that the personal interaction, either with the physician or the study team, successfully compensated for their frustrations and helped alleviate their anger about the confusing study organization. Moreover, patients described the study team as committed to ensuring that the medication arrives at the right time.

One patient mentioned that the support motivated them to participate in the study, stating:

‘I thought that was quite good, Mrs Q (the secretary) picked up […] quite well, that she realized that, these are all people who continue to lead their normal lives and are doing this on top of everything else […] and I thought that was good. (participant 6, standardized group, line 117)

The study's organizational aspects such as filling in the questionnaires and diaries were perceived as extensive paperwork and, at times, impersonal by patients. Some suggested using an online form instead—that would make it easier for the participants and the study team and would mean less paper and organizational difficulties due to the post. Many participants felt that the study's structure—primarily the extensive documentation add a layer of bureaucracy that made it feel “anonymous.” One participant expressed this sentiment, saying:

“It's a relatively anonymous study. And I (…) would (…) describe it by the many colorful questionnaires or (…) protocols that always come.”

*Interviewer*: “You associate that with the study, so to speak?’

*Participant 7*: “Right, so, more paper than person.” (participant 7, placebo group, lines 96–100)

Most patients perceived the questionnaires as well-structured, comprehensible and manageable. While some perceived the questionnaires as annoying and repetitive, others found them interesting to have time to reflect on their symptoms. Nevertheless, it demanded self-structure from the patients as well.

‘(..) I sometimes had to somehow organize myself, when is it time for what and when one questionnaire came before I had even sent the last one, then I had to orient myself briefly—what is it about, which week am I now, what is it now? What exactly am I doing now? (…) so you do have to invest energy, so I think anyone can manage that, but you have to pause every now and then.” (participant 8, placebo group, lines 118–122)

Some participants had unclear expectations regarding the diaries. They expected an open form of daily reflection, allowing for personal input, but instead found them to be more similar to other questionnaires with closed questions and tick boxes. One patient stated:

“Basically, when you write in the diary, you think that you put more prose-like input from yourself in there, and I don't think there was much space for that, or just a few lines. It was okay for me, but I could also imagine that others might want to comment on other levels of health, like mental health or whatever. Maybe a bit more space wouldn't have been bad for that, but for me it was perfectly okay” (participant 6, standardized group, line 91)

### 3.5 Recruitment context and acceptability

The parent RCT faced overall recruitment difficulties, as documented in screening and enrollment logs. In contrast, interviewed participants—i.e., those who enrolled—described high acceptability of study procedures, supportive interactions with the study team, and largely manageable documentation demands. These observations can coexist: barriers to participation operate at the level of reach/demand, while perceived acceptability among enrolled participants remains high. Our qualitative findings therefore speak primarily to *acceptability*—how participants experienced and evaluated study procedures—and *implementation*—how these procedures functioned in practice. They should not be interpreted as evidence against recruitment challenges in the broader sampling frame. In the following, we report acceptability-focused findings and return to implications for implementation and demand in the Discussion.

## 4 Discussion

In this sub-study, we explored the feasibility of the randomized, placebo-controlled HOMEOSAR trial using qualitative methods. This qualitative sub-study primarily addressed two feasibility domains: *acceptability*—how participants experienced and evaluated the procedures—and *implementation*, referring to how study processes functioned in practice. By focusing on interpersonal and organizational mechanisms within these domains, the analysis highlights factors that support or hinder feasibility under triple-blind RCT conditions.

The relationship between patients and physicians appeared to play a central role in perceived feasibility. Patients consistently described their interactions with the physician as positive, with many emphasizing the professional and empathetic approach. These findings align with prior research underscoring the importance of thorough, patient-centered interactions in achieving optimal outcomes as well as the relationship with the physician in homeopathic interventions (25–27, 33). The length and depth of the first contact—often involving detailed biographical exploration—were particularly valued, reinforcing the centrality of individualized care in homeopathy. This process has been described as characteristic of homeopathic treatment (34) and may represent one important element contributing to therapeutic benefit, in line with previous studies highlighting the significance of the consultation process (25–27).

In this light, many of the effects observed in our study could also be interpreted as contextual or placebo-related, with empathy, extended consultation time, and patient expectations acting as important therapeutic mechanisms (25, 27, 33, 35). Brien et al. (25) attributed therapeutic changes to the homeopathic consultation process. In their study, patients receiving homeopathic consultations showed statistically and clinically significant improvements in physical complaints, suggesting that the consultation rather than the remedy accounted for the observed benefits in rheumatoid arthritis. Moreover, Brien et al. (33) discovered that homeopathic consultations supported patients in enhancing their ability to cope, either through improved physical health, overall well-being, or better management of their illness.

Davidson and Jonas (35) suggest that individualized homeopathy integrates aspects of humanistic therapy and narrative medicine, while also utilizing personalized information to guide treatment selection. It may be argued that individualized homeopathy is structured to make optimal use of placebo components, potentially enhancing its effectiveness even in conditions not typically associated with psychotherapy. Furthermore, Geers and Miller (36) argued in their review that non-deceptive intervention techniques can elicit placebo responses. In the context of our study, it is therefore plausible that such placebo and contextual mechanisms, such as empathy, extended consultation time, and supportive study interactions, also contributed to the positive patient experiences reported.

Notably, although logistical challenges such as extensive paperwork and organizational confusion emerged, participants still recognized the support provided by the study team. The well-structured diaries and questionnaires contributed to feasibility and effectively captured patient experiences. Such administrative demands are common in clinical research and can increase participant burden and affect trial feasibility (37, 38). Our findings suggest that such administrative burdens may shape patients' perceptions of implementation feasibility. While these elements occasionally disrupted the physician–patient interaction, they also offered reflective opportunities, potentially enhancing engagement. This dual effect underscores the need to balance methodological rigor with interpersonal connection—particularly relevant in homeopathy, where the therapeutic relationship is likely a key factor for both feasibility and perceived benefit.

Our findings align with established conceptual frameworks of feasibility research. Eldridge et al. (39) distinguish feasibility and pilot studies as preparatory stages for randomized controlled trials, emphasizing their role in assessing whether an intervention can be delivered as intended. Within this broader understanding, the frameworks proposed by Bowen et al. (40) and O'Cathain (30) specify *acceptability* and *implementation* as key domains for evaluating whether interventions are appropriate, practical, and sustainable in real-world settings. Positioning our qualitative substudy within this integrated framework highlights how relational and procedural mechanisms—such as empathy, communication, and organizational clarity—directly influence these domains and inform the design of future patient-centered trials.

By explicitly concentrating on two mechanism-rich feasibility domains—*acceptability* and *implementation*—our analysis yields design-level, transferable insights: how relational work under triple-blind conditions supports participant engagement and how organizational levers (communication, logistics, documentation) can strengthen implementation and organizational capacity. These recommendations extend to patient-centered RCTs beyond CAM (see Supplementary S2 for a summary of feasibility domains and design-level recommendations). Beyond the specific HOMEOSAR context, the findings offer methodological insights for designing patient-centered RCTs in complex intervention settings.

### 4.1 Limitations

This study is subject to selection and self-selection bias: interviewees were generally open to homeopathy and chose to participate in the parent trial. Such predispositions and positive expectations can inflate perceived acceptability or subjective benefit [e.g. (28, 41)], which should temper interpretation of our feasibility findings.

We did not interview decliners or non-participants. This limits insight into demand-side barriers and may bias qualitative accounts toward motivated enrollees. The omission reflects ethical approval and blinding/logistical constraints of the parent RCT. Future feasibility work should incorporate brief refusal-reason capture and an opt-in pathway for follow-up interviews with non-participants.

The qualitative subsample was small and skewed (*n* = 15; 73% male; only two participants in the individualized homeopathy arm), reducing representativeness and constraining cross-arm contrasts. Sampling from within an RCT likely enriched for participants with favorable attitudes toward complementary medicine, which may limit transferability to the broader SAR population.

No formal member checking was conducted, which may reduce credibility. We mitigated this through reflexive team discussions and interdisciplinary analysis to enhance interpretive consistency.

The parent trial experienced recruitment difficulties. These macro-level challenges can coexist with high micro-level acceptability among enrollees; our data speak primarily to the latter. Recruitment occurred during the COVID-19 pandemic, which plausibly affected opportunities for contact (e.g., masking), but participants did not spontaneously mention COVID-19 in interviews, suggesting limited salience for their feasibility perceptions.

Finally, the organizational and documentation burden—especially during the initial case-taking—may have influenced the physician–patient interaction. Study physicians reported that extensive administrative tasks during consultations differed from routine practice and could affect attentional focus and therapeutic presence.

### 4.2 Practical implementation

Findings from this qualitative feasibility substudy highlight several practical considerations for improving *acceptability* and *implementation* in patient-centered RCTs in homeopathy and other complex interventions.

We recommend that future trials ensure a clearly communicated and standardized consultation length, as the duration of case-taking in this study varied considerably due to its individualized approach. Providing participants with contact cards or information sheets listing the names and roles of key study personnel could improve orientation and transparency. In addition, standardized appointment reminders (e.g., via SMS or email) and trackable medication shipments with visible delivery confirmation for participants should be implemented. Particularly for less familiar or controversial treatment approaches such as homeopathy, it is advisable to provide a clear and well-structured written explanation of the study's organizational procedures to proactively reduce uncertainty and prevent confusion during participation.

Because the homeopathic case-taking process is often unfamiliar to participants, a clear organizational framework can provide structure and predictability, helping to offset uncertainty. This is particularly important at the beginning of treatment and during medication administration, when delays or unclear timing may reinforce existing uncertainties about the medication, particularly in the context of a scientifically debated intervention and its mechanisms of action. Transparent communication about the rationale, expected duration, and procedural steps of the case-taking and medication phases can therefore foster trust and enhance acceptability.

Moreover, documentation and data collection processes should be streamlined. Participants perceived paper-based questionnaires as extensive and sometimes impersonal; digital patient-reported outcome tools with limited free-text fields may reduce administrative burden while maintaining opportunities for reflection. Integrating user-friendly digital platforms could also enhance data completeness and timeliness.

Finally, recruitment and retention strategies should be adapted to the context of unfamiliar or time-intensive interventions. Providing flexible scheduling options (e.g., evening appointments or tele-consultations) and a consistent study contact may further enhance accessibility and continuity.

Together, these measures illustrate how targeted organizational improvements can enhance both acceptability and implementation capacity in future feasibility and efficacy trials of homeopathy and comparable patient-centered interventions.

### 4.3 Future research

Future research should continue to address the challenge of the balance between subjectivity and objectivity in homeopathic studies by investigating methods that preserve the therapeutic relationship while ensuring unbiased study conditions. Specifically, the tension between fostering a strong therapeutic alliance with the homeopath (subjectivity) and maintaining the neutrality of clinical study personnel (objectivity) requires innovative methodological approaches. For example, multi-arm or factorial designs can help isolate the effect of the homeopathic consultation from that of the remedy, while mixed-method frameworks integrate quantitative outcome measures with qualitative assessments of the therapeutic process. Additionally, designs such as blinded preference trials or open-label placebo studies may offer strategies to distinguish treatment-specific effects from those arising through contextual or expectation-related mechanisms. Addressing this issue could involve treating the therapeutic relationship as a study variable, while implementing robust measures to ensure unbiased data collection and analysis. Moreover, future research could explore the role of the therapeutic relationship with the physician by comparing treatment groups with and without homeopathic consultations to evaluate its specific impact on outcomes. In a randomized controlled trial, patients could be assigned to a group receiving structured, empathic homeopathic counseling, a group receiving conventional counseling, or a group receiving no counseling, followed by the same placebo medication. The direct and indirect effects of the therapeutic relationship could be assessed using outcomes such as symptom progression, treatment expectations, treatment adherence, and quality of life. This could clarify the importance of the empathic, biographical, and time-intensive physician interaction observed in this study. Future studies should use qualitative methods to investigate reasons for nonparticipation and to explore logistical or perceptual barriers encountered during recruitment—particularly in publicly debated or controversial treatment contexts. Furthermore, reducing the administrative burden through digital questionnaires, collection of only the most important outcomes, and streamlined documentation processes may improve organizational clarity and participant experience, making studies more efficient while maintaining high data quality.

## 5 Conclusion

This qualitative feasibility sub-study explored two key domains of the HOMEOSAR trial—the therapeutic relationship and study organization—to identify design- and process-level factors influencing *acceptability* and *implementation* within a triple-blind randomized controlled setting. Results of the qualitative sub-study indicate the overall feasibility of the HOMEOSAR trial, highlighting the essential importance of the therapeutic relationship between patients and physicians in homeopathic interventions. Patients valued the empathetic and professional care received, with particular emphasis on the depth and attentiveness of the initial consultation. Although the bureaucratic challenges related to documentation posed an additional administrative burden, they may also have encouraged moments of self-reflection. These findings reinforce previous evidence suggesting that the homeopathic consultation process itself may substantially contribute to the overall perceived benefit of treatment. Specific organizational recommendations include clear preparation and communication for participants about the structure and duration of complex or unfamiliar therapies, simplified documentation procedures, and a designated study contact to ensure continuity and adherence throughout the trial.

Future feasibility research should further explore the interplay between relational and procedural elements across homeopathic and conventional study contexts, aiming to optimize both scientific rigor and patient-centered implementation.

## Data Availability

Data is available from Siewert et al. upon reasonable request with restrictions regarding scientific purpose and data protection.
