# Supplementary material for: Feasibility in a homeopathy for seasonal allergic rhinitis RCT: importance of therapeutic relationship and organizational capacity
Source: Front Allergy. 2026 Jan 15;6:1694531. doi: 10.3389/falgy.2025.1694531 (PMC12852988; doi:10.3389/falgy.2025.1694531)
Supplement: Supplementary file 1 [file Table1.docx]

**Supplement S1. Synthesized summaries of the three additional thematic categories**

Supplement S1 provides concise, synthesized summaries of the thematic categories that were not analysed in depth in the main text (motivation to participate, expectations of the medication, perceived effects). These summaries complement the two mechanism-rich feasibility domains presented in the Results section.

| **Theme** | **Summary of key content** |
| --- | --- |
| **Motivation to participate** | Participants’ reasons for joining the trial included being personally affected by seasonal allergic rhinitis, curiosity about homeopathic research, previous negative experiences with conventional medicine, and altruistic motives such as supporting science or helping others. A small number also mentioned financial compensation as a secondary incentive. |
| **Expectation of the medication** | Expectations were moderate and shaped by prior experience with homeopathy. Participants generally hoped for symptom relief and fewer side effects compared with conventional medication. Some anticipated a reduction in their use of conventional drugs or valued being treated with globules as a more “natural” option. |
| **Perceived effects** | Reported experiences varied: some participants described subjective symptom improvement or overall well-being, whereas others noted little or no change. Across accounts, participation itself—regular reflection, structured follow-up, and interaction with the study team—was often perceived as beneficial regardless of symptom outcome. |
