# Supplementary material for: Feasibility in a homeopathy for seasonal allergic rhinitis RCT: importance of therapeutic relationship and organizational capacity
Source: Front Allergy. 2026 Jan 15;6:1694531. doi: 10.3389/falgy.2025.1694531 (PMC12852988; doi:10.3389/falgy.2025.1694531)
Supplement: Supplementary file 2 [file Table2.docx]

**Supplement S2: Practical recommendations across feasibility domains**

Derived from the qualitative feasibility focus (therapeutic relationship; study organization/personal contact).

| Feasibility domain | Evidence from interviews (summary) | Transferable design recommendation |
| --- | --- | --- |
| Acceptability | Empathy and trustful atmosphere valued; consultations perceived as time‑intensive; biographical depth and attentive listening were appreciated. | Communicate consultation length upfront; protect uninterrupted consultation time; provide a brief pre‑visit primer on what the case‑taking entails; offer short check‑ins between visits if needed. |
| Implementation | Occasional confusion about appointments/locations and how to complete diaries; paper-based documentation perceived as impersonal or cumbersome by some. | Centralised appointment reminders (SMS/email) with clear directions; concise diary instructions with an example entry; option for digital ePRO/diary (including a free‑text field) where feasible. |
| Organizational capacity | Paperwork viewed as heavy yet manageable with support; accessibility of the study team helped offset frustrations. | Streamline and merge forms; reduce mailings; assign a single point of contact/helpdesk; use staggered reminders for key milestones (appointments, diary return, rescue‑medication logging). |
