# Supplementary material for: Feasibility in a homeopathy for seasonal allergic rhinitis RCT: importance of therapeutic relationship and organizational capacity
Source: Front Allergy. 2026 Jan 15;6:1694531. doi: 10.3389/falgy.2025.1694531 (PMC12852988; doi:10.3389/falgy.2025.1694531)
Supplement: Supplementary file 3 [file Supplementaryfile1.docx]

Appendix

| **Appendix 1**: Interview Guide  **Questions**   1. **Opening**   I am very pleased that you have agreed to be interviewed as part of this study. Do you have any questions before we begin?   1. **Introductory questions**   How are you currently feeling with your hay fever?  How does hay fever affect your everyday life?   1. **Experience of the treatment**   You are now taking part in our study. How have you found the homeopathic treatment so far?  Have you noticed any changes in your symptoms?  Have you noticed any effects of the treatment on your life and well-being in general?   1. **Start of the study - expectations and quality of life**   Thinking back to the beginning of the study, what expectations and wishes did you have regarding your treatment?  Did you or anyone around you already have experience with homeopathic treatments?  If we stay with the beginning of the study: How did you feel about your hay fever then?  How did you feel last spring?   1. **Relationship to the physician**   Thinking back, please describe your experience of your first consultation with your homeopathic physician.  How did you feel during your consultation with your physician?  What did you like / dislike about the conversation with your physician?   1. **Study**   How do you feel about taking part in this study?  What do you think of the questionnaires?  How did you find completing the diaries?  Looking back, can you please describe why you took part in the study?  Which of the three therapy groups do you think you might have been assigned to?  Why?   1. **Closing**   Is there anything else important from your point of view that we have not discussed so far?  Do you have any questions for me? |
| --- |
